# An increased bone mineral density is an adverse prognostic factor in patients with systemic mastocytosis

**DOI:** 10.1007/s00432-019-03119-3

**Published:** 2020-01-24

**Authors:** Philipp Riffel, Juliana Schwaab, Christopher Lutz, Nicole Naumann, Georgia Metzgeroth, Alice Fabarius, Stefan O. Schoenberg, Wolf-Karsten Hofmann, Peter Valent, Andreas Reiter, Mohamad Jawhar

**Affiliations:** 1 Institute of Clinical Radiology and Nuclear Medicine, University Hospital Mannheim, Heidelberg University, Mannheim, Germany; 2 Department of Hematology and Oncology, University Hospital Mannheim, Heidelberg University, Mannheim, Germany; 3 Department of Internal Medicine I, Division of Hematology and Hemostaseology, and Ludwig Boltzmann Institute for Hematology and Oncology, Medical University of Vienna, Vienna, Austria

**Keywords:** Advanced systemic mastocytosis, Indolent systemic mastocytosis, Bone mineral density, Osteosclerosis, Osteoporosis, Prognosis

## Abstract

**Purpose:**

Systemic mastocytosis (SM) is characterized by the expansion of clonal mast cells that infiltrate various organ systems. The extent of organ infiltration and subsequent organ damage distinguishes between indolent SM (ISM) defined by a nearly normal life expectancy and advanced SM (AdvSM) defined by poor prognosis. In ISM, measurement of the bone mineral density (BMD) frequently reveals osteoporosis. In contrast, the clinical implication of an increased BMD and osteosclerosis remains unclear.

**Methods:**

BMD was evaluated in 61 patients with mastocytosis (ISM, *n* = 29, 48%; AdvSM, *n* = 32, 52%). We correlated the prevalence of osteoporosis, increased BMD and osteosclerosis with clinical parameters, disease variant and prognosis.

**Results:**

Osteoporosis was detected in 11/29 (38%) patients with ISM but only in 2/32 (6%) patients with AdvSM (*p* = 0.004). An increased BMD was detected in 1/29 (3%) patients with ISM and 24/32 (75%) patients with AdvSM (*p* < 0.001) while osteosclerosis was only detected in AdvSM patients (16/32, 50%). AdvSM patients with increased BMD had higher levels of bone marrow mast cell infiltration, higher serum tryptase and alkaline phosphatase levels compared to ISM as well as higher number of high-molecular risk mutations (*p* < 0.05). In addition, we found that the prognosis of AdvSM patients with increased BMD is inferior compared to those without increased BMD (median overall survival 3.6 years versus not reached, *p* = 0.031).

**Conclusions:**

Osteoporosis is a common feature in ISM but not in AdvSM. An increased BMD is frequently present in AdvSM but not in ISM and is associated with more advanced disease and inferior outcome.

## Introduction

Systemic mastocytosis (SM) is a rare hematological neoplasm characterized by the expansion of clonal mast cells that infiltrate various organ systems, e.g., bone marrow, liver, spleen, gastrointestinal tract, lymph nodes and skin (Horny et al. 1985; Valent et al. 2001, 2003; Metcalfe 2008; Jawhar et al. 2015). According to the World Health Organization (WHO), the extent of organ infiltration and subsequent organ damage distinguishes between indolent SM (ISM) and advanced SM (AdvSM). AdvSM comprises patients with SM and an associated hematologic neoplasm (SM-AHN), aggressive SM (ASM) and mast cell leukemia (MCL) (Arber et al. 2016).

ISM patients usually present with a low mast cell burden and have a nearly normal life expectancy while AdvSM patients usually have a high disease burden, multiple-organ damage and poor prognosis with median overall survival of approximately 3–4 years (Jawhar et al. 2016, 2017a, b, 2019; Valent et al. 2017; Sperr et al. 2019).

Because bone and bone marrow (BM) involvement is a characteristic and frequent feature in SM patients, measurement of the bone mineral density (BMD) plays an important role in the diagnostic workup. According to the literature, the prevalence of osteoporosis (*T* score of ≤  − 2.5 standard deviation, SD) in ISM patients ranges between 14 and 66% (Harvey et al. 1989; Floman and Amir 1991; Brumsen et al. 2002b; Kushnir-Sukhov et al. 2006; Donker et al. 2008; Mathew et al. 2009; Rossini et al. 2011). However, the association between SM subtypes and increased BMD or osteosclerosis is yet unknown.

We, therefore, sought to evaluate the prevalence of osteoporosis, increased BMD and osteosclerosis and their impact on clinical characteristics and prognosis in a large cohort of well-characterized patients with various subtypes of SM.

## Materials and methods

In this retrospective analysis, a total of 61 patients (ISM, *n* = 29, 48%; AdvSM, *n* = 32, 52%) were included within our German Registry on Disorders of Eosinophils and Mast Cells.

All patients were scanned on a 16 row CT Scanner (SOMATOM Emotion 16, Siemens Healthcare Sector, Forchheim, Germany). Scans of L1, L2 and L3 were conducted using the following protocol: 120 kV, 250 mAs, and slice thickness 10 mm. Additionally, an in-scan calibration phantom (Siemens OSTEO phantom) was placed beneath the spine during the image acquisition to convert CT values to BMD. The study design adhered to the tenets of the Declaration of Helsinki and was approved by the institutional review board of the Medical Faculty of Mannheim, Heidelberg University (Heidelberg, Germany). All patients gave written informed consent.

The results were expressed as *T* score (standard deviation, SD below the mean of young healthy adults) and as *Z* score (SD below the age- and gender-matched mean reference value). According to established WHO criteria, osteoporosis was defined as a lumbar spine *T* score of ≤  − 2.5 SD (Kanis 1994). Increased BMD and osteosclerosis were defined as *Z* score > 1 SD and > 2 SD, respectively. Because the median age of our cohort was high and following the guidelines of the International Society for Clinical Bone Densitometry (ISCD), we applied the *Z* score for diagnostic purposes of osteoporosis (Rossini et al. 2011). Severe mastocytosis-related osteopenia was defined as a lumbar spine *Z* score of < − 2 SD (Valent et al. 2007).

All statistical analyses considered clinical and laboratory parameters obtained at time of imaging. Overall survival (OS) was measured from the date of diagnosis to date of death or last visit. OS probabilities were estimated with the Kaplan–Meier method and compared by the log-rank test in univariate analysis. The Wilcoxon–Mann–Whitney *U* test was used to compare continuous variables and medians of distributions. For categorical variables, two patient groups were compared with the Fisher’s exact test. All tests were two sided, retaining *p* < 0.05 as statistically significant.

## Results

### Disease characteristics

Patients’ characteristics are listed in Table 1. The male:female ratio was 2:1. Significant differences between ISM and AdvSM included age (median 42 vs. 70 years, *p* < 0.0001), hemoglobin (median 14.1 g/dL versus 10.9 g/dL), platelets (263 × 10^9^/L vs. 114 × 10^9^/L), BM mast cell infiltration (median 10% vs. 30%), serum tryptase levels (36 µg/L vs. 211 µg/L), alkaline phosphatase (median 70 U/L vs. 190 U/L), frequency of splenomegaly (14% versus 94%) and overall survival (OS, median not reached vs. 3.8 years, *p* < 0.0001).

**Table 1:** Baseline clinical, laboratory, genetic, and outcome characteristics of patients with indolent and advanced systemic mastocytosis

Characteristics	ISM (*n* = 29)	AdvSM (*n* = 32)	*P*
Age (years)			
Median	42	70	< 0.0001
Range	28–74	28–82	
Sex, *n* (%)			
Men	12 (41)	21 (66)	NS
Women	17 (59)	11 (34)	NS
Hemoglobin (g/dL)			
Median	14.1	10.9	< 0.0001
Range	12–15.8	7.5–14,8	
< 10 g/dL, *n* (%)	0 (0)	13 (41)	< 0.0001
Platelets (× 10^9^/L)			
Median	263	114	< 0.0001
Range	124–497	39–577	
< 100 × 10^9^/L, *n* (%)	0 (0)	13 (41)	< 0.0001
Mast cell infiltration in BM histology (%)			
Median	10	30	0.0003
Range	5–40	5–95	
Serum tryptase (µg/L)			
Median	36	211	< 0.0001
Range	8–106	14–1250	
Alkaline phosphatase (U/L)			
Median	70	190	< 0.0001
Range	40–244	55–756	
> UNL, *n* (%)	1 (3)	22 (69)	< 0.0001
Splenomegaly, *n* (%)	4 (14)	30 (94)	< 0.0001
*KIT* D816V positive	26 (90)	31 (97)	NS
Follow-up (years)			
Median	12.1	3.6	
Range	0.1–29.2	0.1–19.3	
Overall survival (years)			
Median	NR	3.8	< 0.0001
95% CI	–	2.9–4.7	
Death, *n* (%)	0 (0)	21 (66)	

*AdvSM* advanced systemic mastocytosis, *BM* bone marrow, *CI* confidence interval, *ISM* indolent systemic mastocytosis, *NR* not reached, *NS* non-significant, *UNL* upper normal limit, *WHO* World Health Organization

### Osteoporosis, mastocytosis-related severe osteopenia, and osteosclerosis

Osteoporosis was detected in 11/29 (38%) patients with ISM but only in 2/32 (6%) patients with AdvSM, respectively (*p* = 0.004), while severe mastocytosis-related osteopenia was only diagnosed in ISM patients (*n* = 3, 10%). A false high BMD due to fractures or vertebroplasty in the lumbar spine was detected in 4/29 (14%) ISM patients. An increased BMD was diagnosed in 1/29 (3%) patient with ISM and 24/32 (75%) patients with AdvSM (*p* < 0.001), respectively, and osteosclerosis only in AdvSM patients (16/32, 50%, *p* < 0.0001, Table 2).

**Table 2:** Frequency of osteoporosis, mastocytosis-related osteoporosis, increased bone mineral density, and osteosclerosis in patients with indolent and advanced systemic mastocytosis SM

Characteristics	ISM (*n* = 29)	AdvSM (*n* = 32)	*P*
Osteoporosis^a^, *n* (%)	11 (38)	2 (6)	0.004
Mastocytosis-related osteoporosis^b^, *n* (%)	3 (10)	0 (0)	0.1
Increased BMD^c^, *n* (%)	1 (3)	24 (75)	< 0.001
Osteosclerosis^d^, *n* (%)	0 (0)	16 (50)	< 0.0001

*AdvSM* advanced systemic mastocytosis, *BMD* bone mineral density, *ISM* indolent systemic mastocytosis

^a^*T* score of ≤ − 2.5 standard deviation (SD)

^b^*Z* score < − 2 SD

^c^*Z* score > 1 SD

^d^*Z* score > 2 SD

### Comparison of ISM patients with and without osteoporosis

BM mast cell infiltration (median 10% versus 20%, *p* = 0.035) and serum tryptase levels (median 31 µg/L versus 58 µg/L, *p* = 0.047) were significantly lower in ISM patients with osteoporosis (*n* = 11; 38%) as compared to those without osteoporosis (*n* = 18, 62%) (Table 3).

**Table 3:** Clinical and laboratory characteristics of patients with indolent systemic mastocytosis with and without osteoporosis

Characteristics	No osteoporosis (*n* = 18)	Osteoporosis (*n* = 11)	*P*
Age (years)			
Median	41	44	NS
Range	28–66	32–74	
Sex, *n* (%)			
Men	8 (44)	5 (45)	NS
Women	10 (56)	6 (55)	NS
Mast cell infiltration in BM histology (%)			
Median	20	10	0.035
Range	5–40	5–30	
Serum tryptase (µg/L)			
Median	58	31	0.047
Range	8–106	10–83	

*BM* bone marrow

**Fig. 1:**
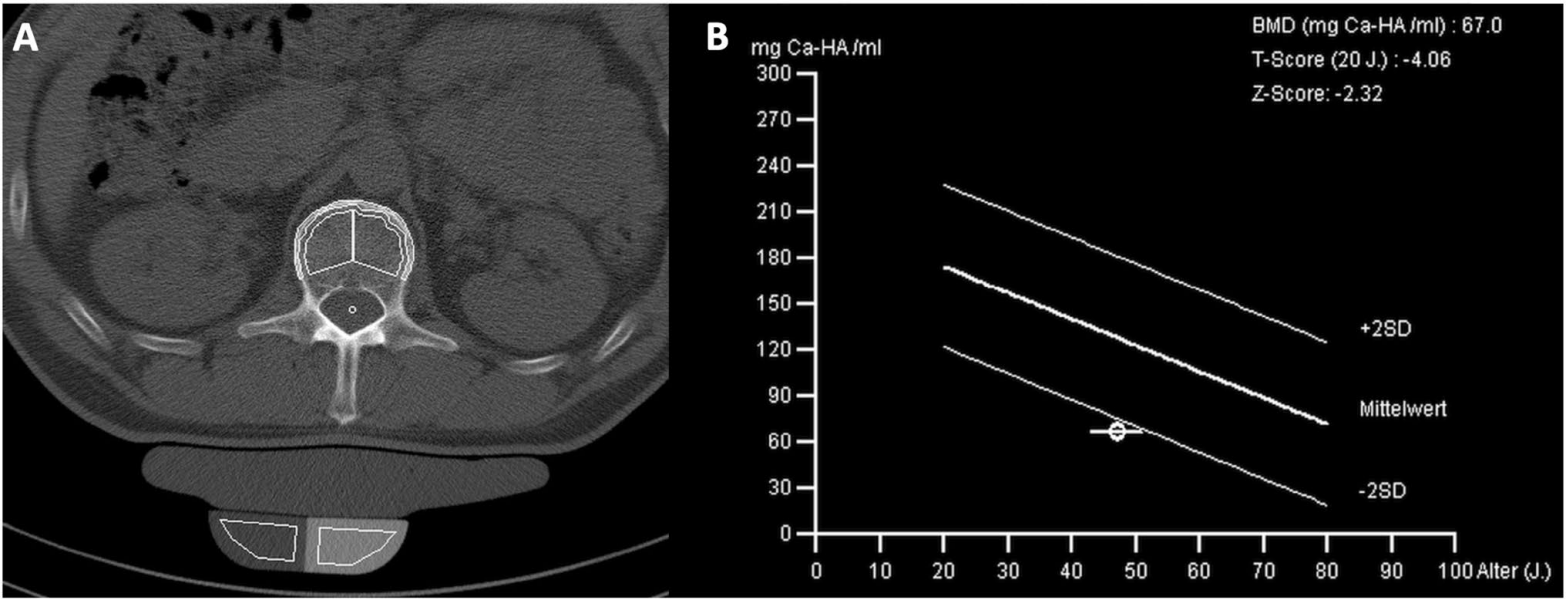
**a** Computed tomography scan of L1 and **b** bone mineral density (BMD) in a 56-year-old male patient with indolent systemic mastocytosis (ISM) and severe osteoporosis according to the traditional WHO criteria (*T* score: − 4.06). Also, a mastocytosis-related low BMD is present with a *Z* score of − 2.32

No significant differences were seen regarding age, gender, blood counts, and OS. Three of 29 (10%) ISM patients had severe mastocytosis-related osteopenia (*T* score < − 2) (Fig. 1).

### Comparison of AdvSM patients with increased and normal BMD

**Fig. 2:**
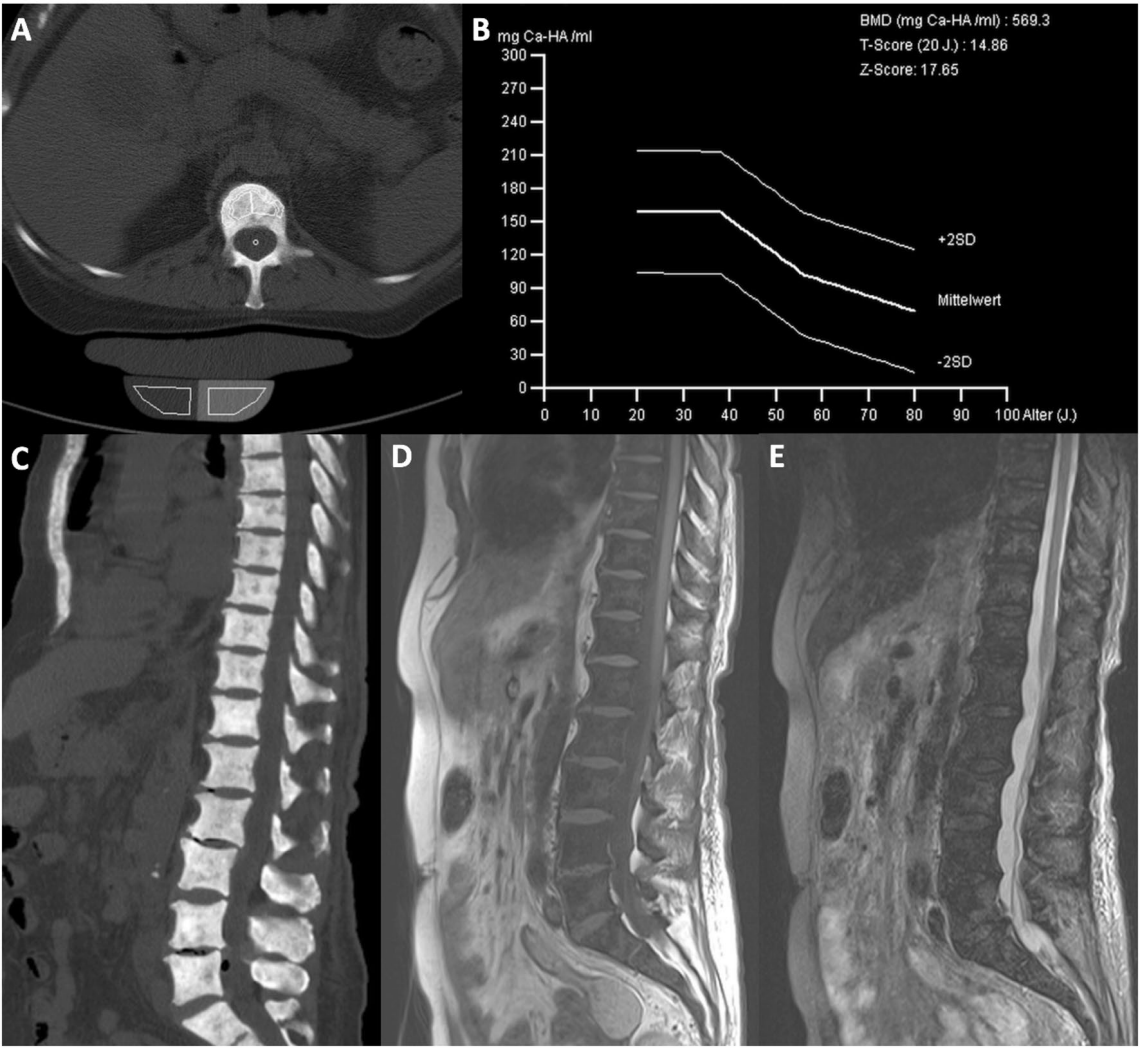
**a** Computed tomography (CT) scan of L1 and **b** bone mineral density (BMD) in a 77-year-old male patient with advanced systemic mastocytosis (AdvSM). Severe osteosclerosis is reflected in the *Z* score of 17.65 and in the corresponding CT scan of the spine (**c**). Also, magnetic resonance imaging (MRI) images of the spine showed diffuse osteosclerosis with a very low signal both in T1-weighted (**d**) and turbo inversion recovery magnitude (TIRM)-sequences (**e**)

**Fig. 3:**
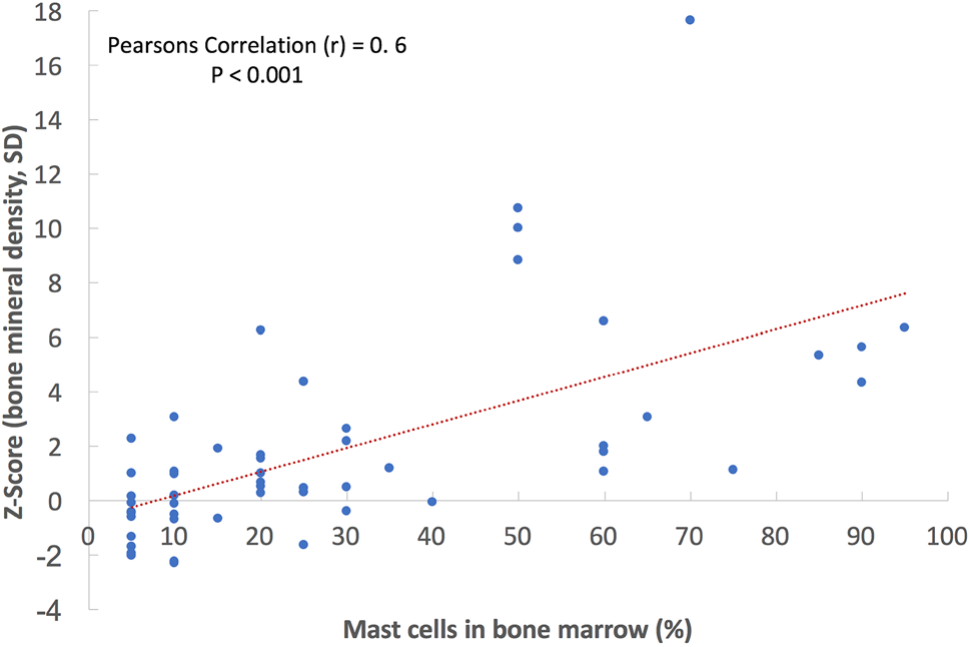
Correlation between bone mineral density and mast cell infiltration in bone marrow histology in patients with systemic mastocytosis

**Fig. 4:**
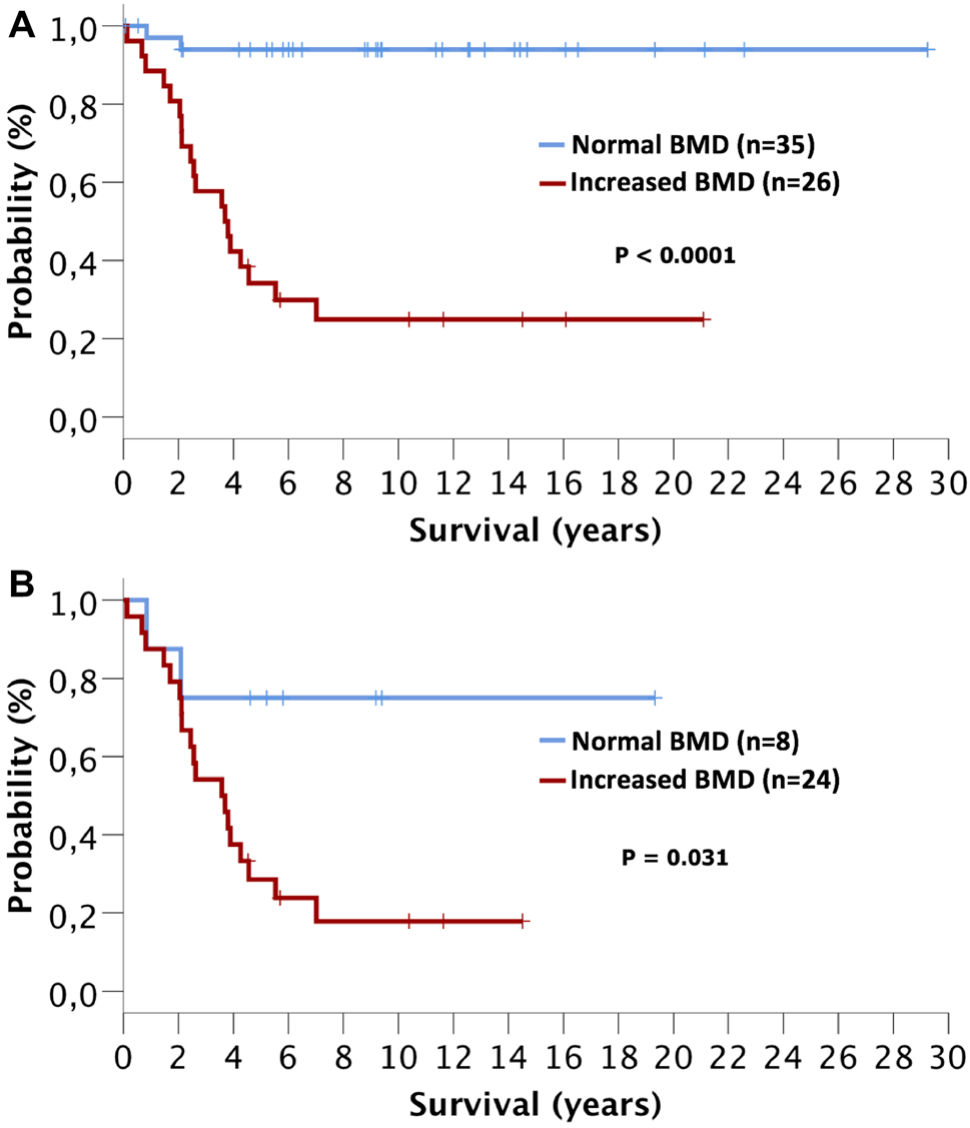
Overall survival of systemic mastocytosis (SM) patients with normal or increased bone mineral density (BMD). **a** Patients with indolent SM and advanced SM (AdvSM) and **b** patients with AdvSM

In comparison to AdvSM patients with normal BMD (*n* = 8, 25%), AdvSM patients with an increased BMD (*n* = 24, 75%, Fig. 2) were older (median 77 years vs. 68 years, *p* = 0.0043) and had lower platelet counts (median 111 × 10^9^/L vs. 238 × 10^9^/L, *p* = 0.041). AdvSM patients with an increased BMD presented with significantly higher levels of BM mast cell infiltration (median 50% vs. 10%, *p* = 0.002) (Table 4, Fig. 3), significantly higher serum tryptase levels (median 262 µg/L versus 62 µg/L, *p* = 0.003), significantly higher alkaline phosphatase levels (median 238 U/L versus 74 U/L, *p* < 0.0001), a higher *KIT* D816V allele burden (median 30% versus 4.3%, *p* = 0.046), higher number of mutations in the high-molecular risk panel (*SRSF2*, *ASXL1*, *RUNX1*, S/A/R gene panel), and an inferior survival (median 3.6 years versus not reached, *p* = 0.031) (Fig. 4). No significant differences regarding clinical/histological/laboratory and genetic characteristics were seen between patients with an increased BMD and those which were even classified as osteosclerosis (data not shown).

**Table 4:** Clinical, laboratory, genetic, and outcome characteristics of patients with advanced SM with normal and increased bone mineral density

Characteristics	Increased BMD (*n* = 24)	Normal BMD (*n* = 8)	*P*
Age (years)			
Median	77	68	0.043
Range	53–81	28–82	
Sex, *n* (%)			
Men	15 (63)	6 (75)	NS
Women	9 (37)	2 (25)	NS
WHO classification			
ASM, *n* (%)	3 (12)	1 (12)	NS
SM-AHN, *n* (%)	17 (71)	7 (88)	NS
MCL ± AHN, *n* (%)	4 (17)	0	NS
Hemoglobin (g/dL)			
Median	10.5	12.9	NS
Range	7.9–14.2	7.5–14.8	
< 10 g/dL, *n* (%)	11 (46)	2 (25)	NS
Platelets (× 10^9^/L)			
Median	111	238	0.041
Range	39–312	45–577	
< 100 × 10^9^/L, *n* (%)	11 (46)	2 (25)	NS
Mast cell infiltration in BM histology (%)			
Median	50	10	0.002
Range	5–95	5–30	
Serum tryptase (µg/L)			
Median	262	62	0.003
Range	30–1250	14–230	
Alkaline phosphatase^a^ (U/L)			
Median	238	74	< 0.0001
Range	76–756	55–173	
> UNL, *n* (%)	21 (88)	1 (13)	< 0.001
*KIT* D816V EAB (%)			
Median	30	4.3	0.046
Range	1–50	1–64	
S/A/R mutation(s)			
1 mutation, *n* (%)	19 (75)	4 (50)	NS
≥ 2 mutations, *n* (%)	10 (42)	0	0.035
Follow-up (years)			
Median	3.6	5.5	NS
Range	0.1–14.5	0.8–19.3	
Overall survival (years)			
Median	3.6	NR	0.031
95% CI	2.1–5.1		
Death, *n* (%)	19 (79)	2 (25)	

*AdvSM* advanced systemic mastocytosis; *AHN* associated hematologic neoplasm; *ASM* aggressive SM; *BM* bone marrow; *BMD* bone mineral density; *CI* confidence interval; *EAB* expressed allele burden; *MCL* mast cell leukemia; *NR* not reached; *NS* non-significant; *S/A/R* one or more gene mutations in *SRSF2*, *ASXL1*, *RUNX1*; *UNL* upper normal limit

^a^The comparison between normal BMD and increased BMD regarding median surivval

## Discussion

So far, only little is known about differences in the prevalence of decreased or increased BMD in the various SM subtypes finally leading to the diagnosis of osteoporosis or osteosclerosis. In addition, the impact of an altered BMD on complications and outcome remains largely unknown (Huang et al. 1987; Johansson et al. 1996; Barete et al. 2010; Manara et al. 2010; Rossini et al. 2011). We, therefore, evaluated *T* and *Z* scores in a larger number of patients with ISM and AdvSM.

Osteoporosis is a typical feature of ISM and has been attributed to the local release of mediators such as histamine, heparin or mast cell-derived proteases (Harvey et al. 1989; Floman and Amir 1991; Brumsen et al. 2002a; Kushnir-Sukhov et al. 2006; Donker et al. 2008; Mathew et al. 2009). In line with previous reports (Harvey et al. 1989; Floman and Amir 1991; Brumsen et al. 2002b; Kushnir-Sukhov et al. 2006; Donker et al. 2008; Mathew et al. 2009; Rossini et al. 2011), osteoporosis was detected in 38% of our patients with ISM but in only 6% of the cases with AdvSM. According to Rossini et al., a *Z* score < 2.0 indicating mastocytosis-related severe osteopenia was identified in 10% of our ISM patients. However, another 14% of the ISM patients examined showed false high BMD values due to fractures and/or vertebroplasty in the lumbar spine.

ISM patients with osteoporosis had a significantly lower median mast cell infiltration in the bone marrow and a lower median serum tryptase level as compared to ISM patients without osteoporosis (Johansson et al. 1996; Kushnir-Sukhov et al. 2006; Rossini et al. 2014). A significant positive correlation was observed between serum tryptase levels and *T* score BMD in the subgroup of ISM. Johansson et al. assumed that mast cell mediators stimulating osteoclasts are dominant in patients with a moderate increase of mast cells, while the osteoblast-stimulating effect of histamine prevails in patients with high histamine metabolite excretion (Johansson et al. 1996).

In contrast to ISM, the vast majority of our AdvSM patients had no osteoporosis but an increased BMD or even osteosclerosis. AdvSM patients with increased BMD/osteosclerosis had a more aggressive phenotype, e.g., a higher mast cell burden in the bone marrow compared to our ISM patients, and the same was found to hold true for serum tryptase, *KIT* D816V EAB and alkaline phosphatase. In addition, we found that patients with increased BMD have an inferior outcome as compared to AdvSM patients with normal BMD. Almost all AdvSM patients with a normal BMD had an SM-AHN with a dominating AHN and a relatively low mast cell burden.

Osteosclerosis is observed in a number of hematologic neoplasms and is recognized as an adverse prognostic (Rollison et al. 2008) feature in myeloproliferative neoplasms (MPN) such as primary myelofibrosis, secondary myelofibrosis, polycythemia vera and essential thrombocythemia. Two previous studies with six SM patients have shown that osteosclerosis was associated with more advanced SM subtypes (Kushnir-Sukhov et al. 2006; Barete et al. 2010). In a recent MRI-based study, a diffuse sclerotic bone marrow pattern of the spine was associated with a significantly higher MC burden, organ dysfunction and inferior survival (Riffel et al. 2019).

The pathophysiology of AdvSM-related increased BMD/osteosclerosis is poorly understood. Mast cell-derived mediators and cytokines may exert a direct stimulatory effect on osteoblast recruitment, proliferation, and activity, while tryptase may increase osteoprotegerin, reducing osteoclast activity and favouring osteosclerosis rather than osteoporosis (Johansson et al. 1996; Chiappetta and Gruber 2006; Gregson et al. 2013).

In conclusion, (1) osteoporosis is a common feature in ISM but not in AdvSM, (2) an increased BMD and osteosclerosis are frequently present in AdvSM but not in ISM, (3) in AdvSM, an increased BMD/osteosclerosis is associated with a more aggressive phenotype, high-risk molecular aberrations, and inferior survival.
